# Fluorescence imaging for real-time detection of breast cancer tumors using IV injection of indocyanine green with non-conventional imaging: a systematic review of preclinical and clinical studies of perioperative imaging technologies

**DOI:** 10.1007/s10549-023-07199-1

**Published:** 2024-01-06

**Authors:** C. Florin Pop, Isabelle Veys, Anne Bormans, Denis Larsimont, Gabriel Liberale

**Affiliations:** 1 Department of Surgical Oncology, Institut Jules Bordet, Université Libre de Bruxelles, Rue Meylemeersch 90, 1070 Brussels, Belgium; 2 Institutional Library, Institut Jules Bordet, Université Libre de Bruxelles, Brussels, Belgium; 3 Department of Pathology, Institut Jules Bordet, Université Libre de Bruxelles, Brussels, Belgium

**Keywords:** Fluorescence imaging, Indocyanine green, Breast surgery, ICG, Breast surgical margins, ICG-FI

## Abstract

**Background:**

This review summarizes the available data on the effectiveness of indocyanine green fluorescence imaging (ICG-FI) for real-time detection of breast cancer (BC) tumors with perioperative imaging technologies.

**Methods:**

PubMed and Scopus databases were exhaustively searched for publications on the use of the real-time ICG-FI evaluation of BC tumors with non-conventional breast imaging technologies.

**Results:**

Twenty-three studies were included in this review. ICG-FI has been used for BC tumor identification in 12 orthotopic animal tumor experiences, 4 studies on animal assessment, and for 7 human clinical applications. The BC tumor-to-background ratio (TBR) was 1.1–8.5 in orthotopic tumor models and 1.4–3.9 in animal experiences.

The detection of primary human BC tumors varied from 40% to 100%. The mean TBR reported for human BC varied from 2.1 to 3.7. In two studies evaluating BC surgical margins, good sensitivity (93.3% and 100%) and specificity (60% and 96%) have been reported, with a negative predictive value of ICG-FI to predict margin involvement intraoperatively of 100% in one study.

**Conclusions:**

The use of ICG-FI as a guiding tool for the real-time identification of BC tumors and for the assessment of tumor boundaries is promising. There is great variability between the studies with regard to timing and dose. Further evidence is needed to assess whether ICG-guided BC surgery may be implemented as a standard of care.

## Introduction

Breast cancer (BC) is the leading cause of cancer in women, affecting approximately 2.3 million women annually and accounting for the highest number of cancer-related deaths [1]. The primary treatment modalities for BC include surgery, hormonotherapy, and/or chemotherapy, depending on tumor characteristics [2]. Successful BC surgery involves achieving complete resection of palpable tumors and the resection of small non-palpable (infra-clinical) tumors with microscopically negative margins, while preserving as much normal breast tissue as possible for optimal aesthetic breast reconstruction [3]. Despite significant advancements in preoperative and intraoperative imaging techniques for improved tumor detection, the rate of positive microscopic margins after conservative BC surgery remains high, ranging from 14.9% to 26% in the literature [3–5].

Precise localization of the breast tumor lesion and the ability to distinguish between malignant and benign tissue during surgery are critical for the successful surgical treatment of BC patients. Various pathological and imaging techniques have been reported for this purpose [5].

Recently, near-infrared (NIR) fluorescence imaging (FI) has emerged as a promising nonionizing imaging technique for detection of cancerous tissue in different clinical conditions, including BC [4, 6, 7]. NIR-FI utilizes light properties in the NIR spectrum (700–900 nm) to image tissue. NIR fluorescence offers advantages such as high tissue penetration (millimeters to centimeters in depth) and low autofluorescence emitted by natural fluorophores in the human body (e.g., porphyrins), enabling good discrimination between tissues containing fluorophores and those that do not, resulting in a high signal-to-noise ratio (SNR) [8, 9].

Several fluorophores that emit in the NIR spectrum have been investigated for BC detection in preclinical and clinical studies, including specific and nonspecific fluorescent probes [4, 8, 10–26]. Specific fluorophores include pegulicianine [16], bevacizumab–IRDye800CW [17], indocyanine green (ICG) in combination with different particles to enhance tumor avidity and specificity, such as human serum albumin [18], *Pseudomonas aeruginosa* azurin peptide p28 [19], low molecular weight heparin (LMWH) ICG-loaded liposomes (LMWH-ICG-Lip) [20], ICG-loaded H-Ferritin (HFn) nanoparticles [21], and functionalized erbium-based rare-earth nanoparticles [22]. However, specific fluorescent probes are currently not approved for clinical use and will not be discussed in this review.

Non-specific fluorophores include methylene blue (MB) [24, 25], 5-aminolevulinic acid (5-ALA) [26], and ICG. Among these, ICG is the most popular and widely used fluorophore. ICG is a water-soluble amphiphilic tricarbocyanine with a molecular weight of 775 Da and a hydrodynamic diameter of 1.2 nm, making it an excellent vascular and lymphatic contrast agent when injected intravenously (IV) or into the lymphatic system via subcutaneous injection. Initially developed for photography during World War II, ICG was later utilized for determining cardiac output, hepatic function, and ophthalmic perfusion. Its rapid Food and Drug Administration (FDA) registration was attributed to favorable characteristics, including confinement to the vascular compartment through binding to plasma proteins, fast and almost exclusive excretion into the bile, and low toxicity. In the 1970s, it was discovered that protein-bound ICG emitted fluorescence under illumination with NIR light (750–810 nm), peaking at around 840 nm [27, 28]. ICG is approved by the FDA and the European Medicines Agency (EMA) for clinical applications as a vascular contrast agent. Due to its safety, affordability, and availability, ICG has become the foundation of NIR-FI for tumor detection [29]. Intraoperative indocyanine green fluorescence imaging (ICG-FI) navigation has emerged as a promising technique for detecting cancerous tissue, including liver, colon, ovarian, head and neck, lung, and breast tumors, enabling surgeons to customize surgery based on real-time intraoperative imaging findings.

Since the first successful detection of BC tumors using ICG-FI reported by Ntziachristos and colleagues in the early 2000s, numerous preclinical and a few clinical pilot studies have demonstrated the detectability of BC tumors using ICG-FI after ICG IV injection [4, 11, 13–15, 29]. The exact physiological mechanism underlying the preferential uptake of ICG in tumor tissues after intravenous injection is not fully understood. The most plausible hypothesis is the ‘enhanced permeability and retention’ (EPR) effect observed in tumoral tissue due to neoangiogenesis [4, 6, 8, 10, 30]. Following intravenous injection, ICG acts as a macromolecule due to its high binding to plasma proteins. In healthy tissues, macromolecule-bound ICG serves as an excellent contrast agent, remaining in the intravascular compartment. In contrast, according to the EPR effect, these macromolecules are thought to extravasate from abnormal tumor vessels into the malignant tumor’s extracellular space. As the half-life of ICG in blood circulation is 3–5 min, ICG rapidly washes out from the intravascular space. Consequently, under NIR illumination, ICG that has accumulated in tumoral tissue emits a fluorescence signal that can be visualized through 5–10 mm of connective tissue thickness, resulting in the observed hyperfluorescence of tumoral tissue in contrast to surrounding normal tissue [30–34].

The objectives of this study were to conduct a systematic literature review on ICG-FI for real-time detection of BC tumors in preclinical and clinical studies of perioperative imaging technologies and provide a summary of evidence-based data on the effectiveness of ICG-FI in BC.

## Methods

This systematic literature review was conducted following the recommendations established by the Preferred Reporting in Systematic Review and Meta-Analysis (PRISMA).

Inclusion and Exclusion Criteria: The focus of our search was on studies that reported real-time perioperative (ex-vivo and in-vivo) ICG-FI in primary breast malignant tumors. This included the following aspects: (1) identification of primary BC tumors using ICG-FI, (2) evaluation of tumor margins after BC surgery using ICG-FI, (3) assessment of fluorescence intensity in BC tumors, and (4) accuracy of ICG-FI in detecting primary BC. In cases where two papers reported on the same population, only the first published study was included. We included either French or English language papers.

The following topics were excluded from the review: Conventional breast imaging for detection of BC, angiographic characterizations of ICG-FI, such as mastectomy flap and breast reconstructive flap vascularization and visualization, sentinel lymph node (SLN) detection, and infraclinical BC tumor marking. Additionally, editorials, reviews, commentaries, letters, and book chapters were excluded.

Sources and Literature Search: A comprehensive search was conducted in the PubMed and Scopus databases with the assistance of a professional medical librarian. The search encompassed articles published before December 2022. Furthermore, the reference lists of the retained articles were analyzed for additional relevant studies that met the inclusion criteria.

The following MeSH terms were used: “Optical Imaging”, “Indocyanine Green”, “Breast Neoplasms”, “breast neoplasms/surgery”, “Mastectomy” and “mammary neoplasms/animal”. Free search terms included: “breast cancer”, “breast neoplasia”, “breast-conserving surgery”, “mastectomy”, “breast surgery”, “fluorescence imaging”, “ICG”, “residual tumor”, “margins” and “animal”. These terms were used in various combinations.

Screening of Titles, Abstracts, and Full Texts: The titles, abstracts, and full texts of relevant studies were screened against the inclusion and exclusion criteria.

Data Extraction and Categorization: Full-text versions of studies that met the inclusion criteria were obtained for comprehensive assessment. The following data were extracted: year of publication, authors, study design, number of subjects, histological cancer type, technical details of ICG-FI (timing, volume/dose of ICG injection, type of FI system, FI intensity analysis program, depth of detection), and, when reported, the accuracy and/or detection rate.

These data were then analyzed and categorized into two groups based on the stage of the experiment: Preclinical experiences (including orthotopic tumor models and animal studies related to BC) or human clinical applications.

## Results

**Fig. 1:**
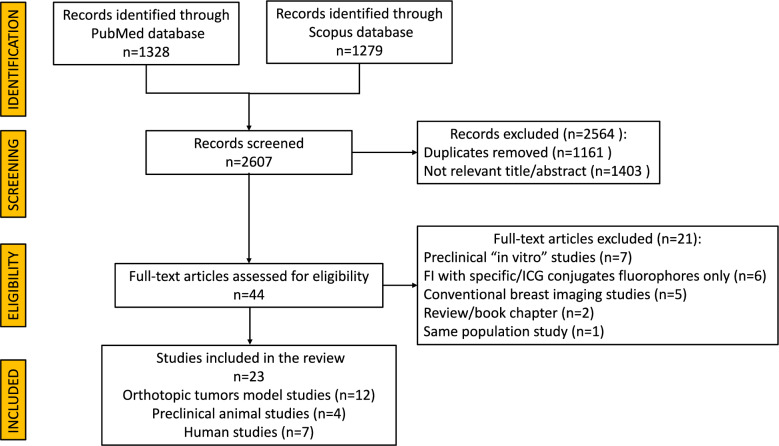
PRISMA flow chart of the study

The search strategy yielded a total of 2607 studies. After removing duplicates, 23 studies published between 1995 and 2022 fulfilled the inclusion criteria and were included in this review. Of these, 16 studies were animal studies, including 12 studies reporting on orthotopic tumor model experiences and 4 studies reporting on preclinical animal assessments. Additionally, 7 studies reported on human clinical applications of ICG-FI. A PRISMA flow diagram illustrating the study selection process is presented in Fig. 1.

### Animal experience in breast cancers with ICG-FI

#### Preclinical orthotopic tumor models for breast cancer tumor detection by ICG-FI

Twelve studies were included in the analysis of ICG-FI detection of orthotopic BC models [10–12, 18–21, 35–39]. Table 1 provides a summary of the characteristics of these studies. The majority of the studies utilized mouse models with subject numbers ranging from 5 to 60. The 4T1-Luc BC cells were predominantly used [10, 12, 18, 20, 21, 35–38]. Seven studies reported both ex-vivo and in-vivo FI, while one study reported only ex-vivo imaging. Various FI systems were employed including, for example, Li et al. and Goto et al. who used the PDE system (Hamamatsu) [11, 19], and Sitia et al. and Sevieri et al. who used the STORZ endoscopic system (Karl Storz Se & Co) [21, 38]. The injected dose of ICG ranged from 0.015 to 7.5 mg/kg. The timing for imaging after ICG IV injection varied across studies, with most studies performing imaging at 24 h post-injection. The detection rate of orthotopic BC tumors ranged from 78% to 100%, and the fluorescence intensity reported as tumor-to-background ratio (TBR) varied from 1.1 to 8.5. Notably, even with short delays in ICG injection (< 8 min and 2 h), the fluorescence intensity remained higher in tumors (TBR at 2.5 and 100 arbitrary units (AU)) [11, 18].

**Table 1:** Studies of ICG-FI in orthotopic models of breast cancer tumors

Author (year)	Animal model (subject no)	Type of BC tumor cells	FI type	Imaging system	Comparison dye	ICG dose	TBR (BC tumor detection)	TBR calculation program	Timing of FI
Li et al. [11] (1995)	Rat (1)	Adenocarcinoma (not specified)	In vivo	PDE (Hamamtsu)	None	0.08 mg/kg^a^ (0.015–0.15 mg/kg)	TBR = 2.5 Detection of very small tumors (0.15 cm^3^)	Formulae calculation	<8 min
Holt et al. [35] (2014)	Mouse (10)	4T1-Luc	In vivo	SpectroPen (LDS system)	None	7.5 mg/kg	Mean TBR = 8.5 Se = 100% (10/10) Detection of 1 mm tumors	Not specified	24 h
Keating et al. [10] (2016)	Mouse (12/60)	4T1-Luc	In vivo and ex vivo	FloCam (BioVision) Artemis FI system (Quest1 Medical Imaging) Iridium (Visionsense)	None	5 mg/kg	TBR = 3.2–4.2 TBR residual tumor = 3.6 Se = 90% (27/30) Sp = 100% NPV = 100%	ImageJ software (NIH, Bethesda)	24 h
Kumar et al. [36] (2017)	Mouse (4)	4T1-Luc and MDA-MB-231	In vivo and ex vivo	Locally developed system	None	0.12 mg/mL	4T1 tumors: CW at 0.8 FLT at 0.99 MDA-MB tumors: CW at 0.97 FLT at 0.99	MATLAB software (MathWorks)	24 h
Lin et al. [18] (2018)	Mouse (5)	4T1-Luc	In vivo and ex vivo	CRi Maestro (UT-SAIR)	HSA-ICG	0.5 mg/kg (100 μL)	100 AU (mean) for ICG vs. 180 AU (mean) for HSA-ICG Se = 100% (5/5) Best timing for the higher ICG intensity at 2 h	NA	10 min and 2–4–8–24 h
Wang et al. [12] (2018)	Mouse (9)	4T1-Luc	In vivo and ex vivo	Locally developed system	None	2 mg/kg^a^ (1–2–4–8 mg/kg)	TBR at 1.78 at 12 h Se = 100% (9/9) 2 mg/kg dose, at times between 12 and 24 h are optimal	Numerical established model	1–2–4–8–12–24–48–72 h
Wojtynek et al. [37] (2020)	Mouse (9)	4T1-Luc	In vivo	FLARE RP1 (Curadel)	Nano-ICG	20 nmol (15.5 mg)	TBR: ICG = 1.96 ± 1.53 Nano ICG = 3.92 ± 2.25 Se = 77.8% (7/9), in the ICG group	ImageJ software (NIH, Bethesda)	24 h
Sitia et al. [38] (2020)	Mouse (6)	4T1-Luc	In vivo	Endoscopic system (KARL STORZ)	HFn-ICG	3.8 mg/kg	NA ICG did not display specific intratumor accumulation at 6 h HFn-ICG showed a strong signal in the tumor at 6 h No fluorescence signals were observed in the tumor in both groups at 24 h	IVIS Lumina II (Perkin Elmer)	6 and 24 h
Sevieri et al. [21] (2021)	Mouse (12)	4T1-Luc	Ex vivo	Endoscopic system (KARL STORZ)	HFn-ICG	3.8 mg/kg	NA The fluorescence intensity in tumors was lower with ICG than with HFn-ICG at 6 and 24 h	IVIS Lumina II (Perkin Elmer)	6 and 24 h
Wang et al. [39] (2021)	Mouse (6)	MDA-MB-231	In vivo and ex vivo	IVIS Lumina II (Perkin Elmer)	HSA-ICG	1 mg/kg	TBR: ICG = 3.87 ± 0.68 Vs. HSA-ICG = 8.19 ± 1.30	MATLAB software (MathWorks) and ImageJ software (NIH, Bethesda)	24 h
Cao et al. [20] (2021)	Mouse (10)	4T1-Luc	In vivo and ex vivo	NA	LMWH-ICG-Lip	100 μg/mL	NA The fluorescence intensity in tumors was 12.57-fold higher with LMWH-ICG-Lip than that of free ICG	Living Image 4.0 (Spectrum)	12 h
Goto et al. [19] (2022)	Mouse (15)	MDA-MB-231	In vivo and ex vivo	PDE Neo (Hamamatsu) Odyssey scanner (LI-COR Biosciences)	ICG-p28	0.5 mg/kg	TBR: ICG = 1.1 Vs. ICG-p28 = 3.5	ImageJ software (NIH, Bethesda)	24 h

*4T1-Luc* murine breast cancer cells, *AU* arbitrary units, *BC* breast cancer, *CW* continuous wave imaging, *FI* fluorescence imaging, *FLT* fluorescence lifetime, *HFn-ICG* H-Ferritin nanoparticles, *HSA-ICG* human serum albumin-assembled indocyanine green nanoprobes, *ICG* indocyanine green, *ICG-p28* indocyanine green—*Pseudomonas aeruginosa* azurin and its derived peptide p28, *LMWH-ICG-Lip* low molecular weight heparin modified indocyanine green-loaded liposomes, *MDA-MB-231* human breast cancer cell line, *NA* not available, *NanoICG* indocyanine green-loaded self-assembled hyaluronic acid nanoparticles, *NIH* National Institutes of Health, *TBR* tumor-to-background ratio

^a^The representative ICG dose in the study

#### ICG-FI and preclinical animal experiences

Four studies were included in this category [13, 14, 40, 41], all conducted on dogs. Table 2 summarizes the principal characteristics of these animal experiences with self-developed BC tumors. The number of subjects varied from 1 to 16, with the evaluation of 1 to 20 malignant breast tumors per dog. All studies employed in vivo evaluation of BC using ICG-FI, with different FI systems utilized. ICG doses ranged from 1 to 5 mg/kg, and the reported TBR of BC varied between 1.3 and 3.9 AU. Two studies utilized the ImageJ software (NIH, Bethesda) for quantification of fluorescence intensity and TBR calculation [14, 41]. Similar to orthotopic model studies, the timing for performing ICG-FI after ICG IV injection varied across studies, ranging from 10–23 min to 48–72 h. Ex-vivo FI identified BC tumors in 24 out of 28 cases, while in-vivo imaging identified tumors in 21 out of 28 cases. The sensitivity of ICG-FI in detecting BC tumors in dogs ranged from 80% to 100%. All three tumors with a short ICG IV injection delay (minutes) were visible by FI [13, 40]. The mean TBR reported for BC tumors in dogs varied from 1.4 to 3.9, with higher values observed in cases with a short ICG injection time (<30 min) [13, 40].

**Table 2:** Summary of animal experiences with ICG-FI for breast cancer tumor detection

Author (year)	No. of subjects	Type of BT	FI type	Imaging system	Exposure time	Working distance	ICG dose	ICG-FI detection depth	TBR (BC tumor detection)	TBR calculation program	Timing of FI
Reynolds et al. [13] (1999)	2 Dogs	Malignant mixed mammary tumor Papillary adenoma	In vivo and ex vivo	Locally developed system	NA	5 cm	1 mg/kg	10–15 mm	TBR > 3	NA	0–23 min and 90–120 min
Gurfinkel et al. [40] (2000)	1 Dog	Adenocarcinoma (not specified)	In vivo	Locally developed system	0.2 s	NA	1 mg/kg	5–10 mm	TBR = 3.9 No significant difference in the ICG uptake rates between normal and diseased tissue regions	MATLAB software (MathWorks)	10–50 min and 48–72 h
Favril et al. [41] (2020)	5 Dogs	3 Adenocarcinoma 1 Adenoma 1 Extraskeletal osteosarcoma	In vivo and ex vivo	Fluobeam 800 (Fluoptics)	5–10–20–40 ms	20 cm	5 mg/kg	NA	In vivo TBR = 1.3 (0.8–1.7) In vivo 2/5 BCT identified with FI Ex vivo TBR = 2.9 (1.4–5.7)	ImageJ software (NIH, Bethesda)	24 h
Newton et al. [14] (2020)	16 Dogs	20 Malignant mammary tumor 21 Benign mammary tumor	In vivo and ex vivo	Solaris (Perkin Elmer)	10 ms	75 cm	3 mg/kg	NA	In vivo Mean TBR = 1.5 (SD 0.2) Se = 80% (16/20) Sp-42.8% NPV = 69.2%	ImageJ software (NIH, Bethesda)	20 h

*BC* breast cancer, *BT* breast tumor, *FI* fluorescence imaging, *ICG* indocyanine green, *TBR* tumor-to-background ratio, *NA* not available, *NIH* National Institutes of Health

### ICG-FI clinical experience in human breast cancers

#### Breast cancer detection

Clinical experiences with ICG-FI in the detection of BC started in 2016 with a study by Keating et al. [10]. Since then, six other studies have been published on human clinical applications of ICG-FI in BC [4, 15, 16, 31–33]. Table 3 summarizes the characteristics of these studies. All studies were pilot studies that aimed to evaluate the feasibility of BC detection using ICG-FI (phase 0–2 studies) and included a limited number of patients, ranging from 8 to 43 patients per study [16, 31]. Patients with both histological adenocarcinoma types, ductal and lobular invasive BC, were included. Histological characteristics of BC were reported in two out of seven studies [4, 31]. Six studies explored primary BC during upfront surgery, while one study investigated neoadjuvant chemotherapy (NAC) [31].

**Table 3:** Human studies of ICG-FI for breast cancer tumor detection and intraoperative margin evaluation

Author (year)	No. of patients	Type of BC tumor	Tumor grade	FI type	Imaging system	ICG dose	Timing of IV ICG injection	Accuracy of ICG-FI	Mean TBR (ex vivo)	TBR (calculation program)	Size of detected lesion
Keating et al.^a^ [10] (2016)	12	IDC = 9 ILC = 3	Not specified	In vivo/ex vivo	FloCam (BioVision) Artemis Fluorescence Imaging system (Quest Medical Imaging) Iridium (Visionsense)	5 mg/kg	24 h before surgery (20.8–27 h)	Se = 100% FPR^a^ = 50%	3.7	ImageJ software (NIH, Bethesda)	7–26 mm
Veys et al. [31] (2018)	8^b^	IDC = 8 ILC = 1	Gr2 = 2 Gr3 = 6	Ex vivo	Fluobeam 800 (Fluoptic)	0.25 mg/kg	47–135 min before surgery	Se = 94.2% Sp = 31.7% NPV = 92.7% FPR = 68%	3.3 (SD 1.7)	IC-Calc 2.0	NA
Pop et al.^a^ [4] (2021)	35	IDC = 32 ILC = 3	Gr1 = 20 Gr2 = 7 Gr3 = 8	In vivo/ex vivo	Fluobeam 800 (Fluoptic)	0.25 mg/kg	20–83 min before surgery	Se = 88.6% (31/35) Se^a^ = 100% Sp^a^ = 60% NPV^a^ = 100 FPR^a^ = 40%	2.4 (1.5)	IC-Calc 2.0	4–40 mm
Bourgeois et al. [15] (2021)	5^c^	NA	Not specified	Ex vivo	PDE (Hamamatsu Photonics)	0.25 mg/kg	24 h before surgery	Se = 40% (2 of 5 patients)	2.7	IC-Calc 2.0	NA (2 mm the small BC tumor foci)
Leiloglou et al. [32] (2021)	10 (5)	IDC = 8 ILC = 1 IMC = 1	Not specified	In vivo/ex vivo	Locally developed system	12 mg/patient	Not specified (< 5 min, vascular phase)	AUC = 0.58–0.88	NA	Matlab software (Mathworks, Inc., Massachusetts, USA)	NA
Kedrzycki et al. [33] (2021)	32 (16 EPR vs 16AP)	IDC = 17 vs. 12 ILC = 1 vs. 3 IMC = 1 vs. 0 IMPC = 0 vs. 1 DCIS = 1 vs. 3	Not specified	Ex vivo	Locally developed system	0.25 mg/kg	25 and 5 min	Se = 72% vs. 85% Sp = 93% vs. 98% NPV = NA FPR = NA	2.1 (0.9) vs. 3.2 (1.7)	Matlab software (Mathworks, Inc., Massachusetts, USA)	1.7–30 mm vs. 0–34 mm
Hwang et al.^a^ [16] (2022)	43	IDC = 36 IMPC = 3 IMC = 2 DCIS = 2	Not specified	In vivo/ex vivo	Real-IGS, (Nuoyuan Medical Equipment)	0.5 mg/kg	2 h before surgery	Se = 100% (43/43) Se^a^ = 93.3% Sp^a^ = 96%	2.14 (0.6)	ImageJ software (NIH, Bethesda)	≤2 cm (67.4%) 2–4 cm (32.6%)

*AUC* area under the curve, *BC* breast cancer, *DCIS* ductal carcinoma in situ, *FPR* false positive rate, *IDC* invasive ductal carcinoma, *ILC* invasive lobular carcinoma, *IMC* invasive mucinous carcinoma, *IMPC* invasive micropapillary carcinoma, *NA* not applicable, *NPV* negative predictive value, *SD* standard deviation, *Se* sensitivity, *Sp* specificity

^a^Intraoperative margin evaluations

^b^Treated by neoadjuvant chemotherapy

^c^Only the results of the groups of patients allocated in the first study protocols

All seven studies included evaluations of ICG-FI using ex-vivo FI, and four studies reported concomitant in-vivo imaging. Different imaging systems were used in each study, except for Veys et al. and Pop et al., who both used the Fluobeam 800 system (Fluoptic, Grenoble, France), and Leiloglou et al. and Kedrzycki et al., who used their own developed system [4, 31–33]. For instance, Keating et al. tested three different FI systems in their 12 patients. The injected dose of ICG for BC detection varied from 0.25 mg/kg (in four studies) to 5 mg/kg (in one study). The timing for imaging after IV injection varied, with some studies starting intraoperatively, as soon as 5 min after ICG injection [32, 33], while others ranged from 20 to 135 min [4, 16, 31, 33], and 24 h in two studies [10, 15]. Different programs, including IC-Calc 2.0, Matlab software (Mathworks, Inc., Massachusetts, USA), and ImageJ software (NIH, Bethesda), were used for quantifying fluorescence intensity and calculating TBR.

Ex-vivo imaging was used in all studies to visualize and identify BC. In the preoperative injection setting, the rate of BC detection varied from 40% in patients injected with a low dose (0.25 mg/kg) of ICG 24 h before imaging to 100% in those injected with a high dose (5 mg/kg) the day before FI [10, 15]. In the intraoperative injection setting, the detection rate of BC varied from 72% to 100% [4, 16, 31–33]. Notably, in one study that used intraoperative ICG injection with an imaging interval shorter than 5 min, the detection rate of BC was 85% [33]. The mean TBR reported for human BC varied from 2.1 to 3.7. In the preoperative injection setting, TBR values of 2.0 and 3.5 were reported in two out of five patients injected with a low dose of ICG the day before surgery, while a mean TBR of 3.7 was reported in those injected with a high dose [10, 15]. In the group of patients injected intraoperatively, the mean TBR reported was homogeneous, ranging from 2.1 to 3.3 [4, 16, 31–33].

#### Margin evaluation

Only three clinical studies explored the use of ICG-FI for evaluating surgical margins in BC [4, 10, 16]. Two different injection time strategies were used: one using ICG injected 24 h before surgery and the other using ICG injected intraoperatively or shortly before surgery (2 h). In a pilot study of 12 patients injected 24 h before surgery with a dose of 5 mg/kg, Keating et al. reported residual fluorescence in the tumor bed in 6 out of 12 patients, but none of these patients had positive margins on definitive pathology [10]. Pop et al., in a pilot study of 35 patients injected intraoperatively with a dose of 0.25 mg/kg, reported a sensitivity (Se), specificity (Sp), and negative predictive value (NPV) of ICG-FI to predict margin involvement on breast operative specimens of 100%, 60%, and 100%, respectively [4]. Recently, Wang and colleagues reported their data on 43 BC patients who were injected with ICG at 0.5 mg/kg, 2 h before surgery, and found an intraoperative sensitivity and specificity for ICG-FI in distinguishing between normal tissue (clean margins) and tumoral tissue (positive margins) of 93.3% and 96.0%, respectively [16].

## Discussion

FI has the potential to be a highly beneficial technique for real-time tumor identification and assessment of tumor boundaries during surgical procedures, particularly in BC. However, its clinical applications for tumor resection are currently limited, with only a few studies conducted in BC [4, 10, 26, 33, 34, 42, 43].

In this systematic review of the literature, 23 studies were included that evaluated the efficacy of ICG-FI for discriminating between benign breast tissue and neoplastic BC. Among these studies, only 7 utilized ICG-FI in clinical settings, and all of them were in the proof-of-concept or feasibility phase [4, 15, 16, 31–33]. The results from these studies show promise for the use of ICG-FI in BC surgery. The detection rate of orthotopic BC tumors and BC tumors in dogs using ICG-FI ranged from 78% to 100% and 80% to 100%, respectively [13, 14, 40, 41]. In clinical experiences, ICG-FI was able to detect tumoral disease in approximately 8 out of 10 women (with a sensitivity between 80% and 100%) when ICG was injected shortly before surgery (within 2 h) [4, 15, 16, 31–33]. The mean TBR reported for BC tumor identification in these studies varied from 1.1 to 8.5, and in human clinical studies, it ranged from 2.1 to 3.7. These TBR values are higher than the threshold detection value (1.3–1.5) by the human eye to define the tissue as hyperfluorescent regardless of cancer type, evaluation type (in vivo or ex vivo), and the FI camera system used [31, 44].

One major challenge in BC surgery is the intraoperative assessment of breast surgical specimens during breast-conserving surgery to rapidly detect residual tumoral disease [3–5]. Early evidence suggests that ICG-FI can be used for the intraoperative evaluation of surgical margin resection after breast conserving surgery, potentially improving surgical treatment outcomes for BC patients [4, 10, 31–34]. With a reported high negative predictive value (100%), ICG-FI examination of the surgical bed may be able to exclude a positive resection margin with certainty, focusing intraoperative pathological evaluation only on cases where residual fluorescence is observed [4, 31].

However, it’s important to interpret these results with caution due to the variability between studies, especially in terms of research stage (preclinical and clinical phase I settings), ICG dose, timing of ICG-FI, FI camera systems, and fluorescence intensity quantifications. More precision is needed regarding the pathophysiological mechanism of action, dosing, and timing of ICG-FI [42–45].

### Mechanism of action of ICG in breast tumors

The mechanism of preferential uptake of ICG in tumor tissues is not fully understood, but likely involves the EPR effect observed in tumor tissue due to abnormal neoangiogenesis. ICG molecules injected intravenously bind to serum lipoproteins and accumulate in the extravascular space of tumor tissue, emitting fluorescence under NIR illumination. The fluorescence signals can be visualized through connective tissue up to 5–10 mm thick. The rapid clearance of ICG from the intravascular space results in the observed hyperfluorescence of tumoral tissue compared to surrounding normal tissue [7–9, 28, 29, 45, 46].

Another lesser-known mechanism of action of ICG in cancer cells, including BC, is its vascular contrast agent properties. During or shortly (3–10 min) after intravenous administration of an ICG bolus, the fluorescence of tumor cells is enhanced due to the binding of ICG to plasma lipoproteins. This mechanism improves the contrast between tumor and normal breast tissue, surpassing pure absorption contrast [7, 28, 29]. Recent studies have shown higher vessel density and increased branch points of the vasculature in breast tumors, which may contribute to the angiographic effect of ICG in breast tumors [47].

A recent study conducted by the group at Imperial College London provides further insight into the mechanism of action of ICG-FI for intraoperative detection of BC tumors [32, 33]. Their findings suggest that the diagnostic accuracy of ICG-FI is improved when the imaging is performed during the angiography phase (within 5 min) compared to longer intervals (over 25 min) after ICG administration. The ex-vivo TBR in the angiography cohort was 3.18 (SD 1.74) compared to 2.10 (SD 0.92) in the later ICG-FI cohort, indicating better tumor detection in the angiography phase [33]. However, it is important to note that larger and adequately powered clinical trials are necessary to confirm these findings.

In addition to the previously discussed pathophysiologic mechanisms of tumor hyperfluorescence, a new pathway for ICG accumulation within tumor cells has been described [48–51]. This mechanism involves passive tumor cell targeting of ICG through increased uptake via clathrin-mediated endocytosis (CME), facilitated by the high endocytic activity of tumor cells and disruption of tight junctions. This phenomenon was initially observed in a mouse model of colorectal cancer [48, 49] and subsequently confirmed in studies involving sarcoma and BC cell lines [50, 51]. It appears that the affinity of ICG for phospholipid components of the cell membrane, which are altered and enriched in tumor cells, contributes to its ability to bind to and pass intracellularly via CME [52]. Furthermore, tumor cells retain the dye for an extended period (at least 24 h) compared to normal tissues, indicating increased cellular uptake and retention as the primary mechanism of tumor fluorescence, rather than solely relying on the EPR effect. These contrasting findings highlight the complexity of the intra-tumor accumulation of ICG, suggesting that multiple mechanisms, including dysregulation of cancer cell pathways, tumor microvasculature, and the EPR effect, likely contribute to its enhanced uptake in tumors. The specific factors at play may vary depending on the tumor type. Further research is needed to better understand the mechanisms of action of ICG at the cellular level within human tumor tissue, which can provide valuable insights for the clinical use of ICG in fluorescence-guided surgery and potentially other diagnostic and treatment applications. This deeper understanding can help optimize aspects such as dosage and timing of ICG administration, which are still not fully elucidated.

### ICG dose and timing

The optimal timing and dose of ICG administration for visualization and delineation of BC tumors through ICG-FI are crucial for accurate diagnosis during surgery. However, reports in the literature vary considerably in terms of protocols and inconsistent findings regarding the effectiveness of different timing and dosing strategies.

Studies have indicated that a low dose of ICG (less than 0.5 mg/kg) administered 24 h before ICG-FI is not effective for BC tumor detection [15, 18, 19]. Even a dose of 1 mg/kg administered the day before surgery does not appear to be sufficient for satisfactory tumor visualization by FI [12, 39, 40]. These findings suggest that preoperative injection, 24 h before surgery, is not the optimal timing for ICG administration.

It is worth noting that most clinical studies, except one, utilized intraoperative ICG injection timing (within ≤120 min) [10]. In contrast, only 4 out of 16 preclinical studies used intraoperative timing. Additionally, studies have explored various injection times ranging from a few minutes to over 24 h, with inconsistent results. However, the interpretation of these findings is challenging due to the variations in ICG dose used across different studies.

Clinical applications of ICG-FI for intraoperative BC detection or discrimination between benign and malignant tissue have mostly been conducted with short delays between ICG injection and FI (ranging from <5 to 143 min) and lower ICG doses (0.25–0.5 mg/kg). These studies reported relatively higher efficacy (sensitivity) ranging from 72% to 100% for BC tumor detection [4, 16, 31–33]. The intraoperative injection timing and lower ICG dose used in these studies make this ICG-FI strategy more easily integrated into current clinical workflows with minimal inconvenience for patients.

It is important to note that, although the literature on the application of ICG-FI in human BC is limited, the existing studies demonstrate significant heterogeneity and variation in reporting the efficacy of ICG-FI. Some studies focus solely on TBR, while others report sensitivity, specificity, negative predictive value, and false-positive rate. To ensure accurate evaluation and comparison of different timing, dosing, and FI systems/strategies, future studies should adhere to reporting complete test accuracy data.

In summary, there is a need for standardized protocols and comprehensive reporting of test accuracy data in future studies on ICG-FI for BC. This will facilitate better comparisons and understanding of the optimal timing and dose of ICG administration, as well as the effectiveness of different FI systems and strategies.

### Imaging systems and FI quantification

We highlight an important limitation in the field of ICG-FI for BC detection, which is the wide variety of FI systems used in both preclinical and clinical settings. In clinical applications alone, seven different FI systems were utilized across seven different studies, with one study even testing three different FI systems in just 12 patients [10]. This variability in FI systems makes it challenging to compare and interpret the results of these early experiences of ICG-FI in BC.

Furthermore, the handheld camera models used for intraoperative imaging may not be well-suited, especially for evaluating the breast surgical cavity after breast-conserving surgery. Although optical imaging systems may have similar characteristics, there is a lack of direct comparison between these systems. Standardization of functionality and results, along with a checklist of performance criteria, should be required and provided by manufacturers to enable meaningful comparisons between different FI cameras. Perhaps certain FI systems need to be adapted for specific uses such as angiographic assessment, sentinel node detection, or evaluation of different types of tumoral tissue. However, currently, there is no recommended device specifically tailored for ICG-FI in BC tumors, and a comparative evaluation of the existing FI systems is necessary to determine the optimal imaging approach [53].

Another challenge in ICG-FI is the quantification of fluorescence intensity, which further complicates result interpretation and comparison. This issue is not limited to BC tumor detection but is also relevant for assessing tissue viability through vascular assessment [54–56]. In the reviewed studies, fluorescence signal quantification and TBR calculations were performed using three different programs across six out of the seven clinical studies that included quantification [4, 10, 15, 16, 31, 33]. Multiple programs with varying algorithms are being evaluated, but efforts should be made by manufacturing companies to develop quantitative imaging systems that are user-friendly and can facilitate the clinical implementation of intraoperative FI in various indications [54].

### Perspectives and limitations

Despite the heterogeneity in ICG dose, timing, and fluorescence systems used in preclinical and clinical evaluations of BC tumors with ICG-FI, the results of the few clinical studies available appear promising [4, 16, 31, 33]. However, future prospective controlled studies are still needed to better define the optimal timing and ICG doses for ICG-FI and to strengthen the current evidence supporting its use in guiding BC surgery in clinical practice.

ICG-FI for BC tumor-guided surgery offers several advantages, including the relatively low cost of the fluorescent dye and its safety for patients and the medical team as a non-invasive agent [57].

It is important to address some limitations of the present review. First, the level of evidence for the results obtained and presented in this review is low due to the current literature, which primarily consists of case series with a small number of patients and considerable heterogeneity. Second, the review could not provide clear-cut results regarding the optimal dose and timing of ICG injection for ICG-FI in BC tumor evaluation. One reason for this is the lack of comparison or control groups in clinical studies. The implementation of ICG-FI in BC tumor detection does not seem to follow a reliable translational approach, as most preclinical studies use a high dose of ICG and a preoperative timing of 24 h, while most clinical trials employ low doses and short intervals until FI. This difference may be attributed to the authors’ tendency to use shorter intervals between ICG injection and surgery to better align with clinical settings. Additionally, the discrepant results between preclinical and clinical studies may be explained by differences in the accumulation, distribution, and persistence of fluorescence signals after ICG IV injections between animal orthotopic tumors and true human tumors.

## Conclusions and future directions

This systematic review of the literature is the first summarizing the results of ICG-FI in BC surgical procedures. Our findings demonstrate promising evidence that detection of BC tumoral tissue and tumor-margin delineation can be improved in clinical practice with the use of ICG-FI as an adjunctive real-time tool. ICG-FI for BC tumor-guided surgery can offer several complementary advantages, such as the relatively low cost of the fluorescent dye and its safety for the patient and the medical team. The variety of FI systems used in perioperative ICG-FI for BC detection, along with the lack of standardized functionality, result reporting, and quantification methods, poses challenges for comparing and interpreting results. Standardization efforts and comparative evaluations are needed to identify the most suitable FI system and establish consistent quantification approaches. While the current evidence on ICG-FI for BC tumor detection shows promise, further well-designed prospective controlled studies are needed to determine the optimal dose and timing of ICG injection. This will provide stronger evidence to support the clinical use of ICG-FI in BC surgery.

## Data Availability

Not applicable
